# Mechanoenzymatic reactions for the hydrolysis of PET†

**DOI:** 10.1039/d3ra01708g

**Published:** 2023-03-29

**Authors:** Esther Ambrose-Dempster, Leona Leipold, Dragana Dobrijevic, Maria Bawn, Eve M. Carter, Gorjan Stojanovski, Tom D. Sheppard, Jack W. E. Jeffries, John M. Ward, Helen C. Hailes

**Affiliations:** a Department of Chemistry, University College London 20 Gordon Street London WC1H 0AJ UK h.c.hailes@ucl.ac.uk; b Department of Biochemical Engineering, University College London Bernard Katz Building, Gower Street London WC1E 6BT UK

## Abstract

Recent advances in the enzymatic degradation of poly(ethylene terphthalate) (PET) have led to a number of PET hydrolytic enzymes and mutants being developed. With the amount of PET building up in the natural world, there is a pressing need to develop scalable methods of breaking down the polymer into its monomers for recycling or other uses. Mechanoenzymatic reactions have gained traction recently as a green and efficient alternative to traditional biocatalytic reactions. For the first time we report increased yields of PET degradation by whole cell PETase enzymes by up to 27-fold by utilising ball milling cycles of reactive aging, when compared with typical solution-based reactions. This methodology leads to up to a 2600-fold decrease in the solvent required when compared with other leading degradation reactions in the field and a 30-fold decrease in comparison to reported industrial scale PET hydrolysis reactions.

## Abstract 2

Mechanoenzymatic reactions are described for the degradation of different PET materials using whole cell PETases.

## Graphical abstract

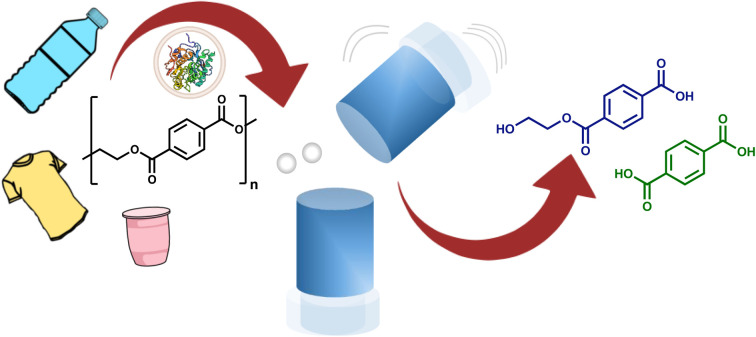

## Introduction

Global plastic production reached 460 million tonnes in 2019,^1^ with PET constituting approximately 10% of this value,^2^ which because of its resistance to degradation, has led to its accumulation in the environment. The production of PET is projected to increase annually, and current thermomechanical methods of recycling reduce the quality of the plastic, limiting post-recycling applications, whilst chemical degradation of PET requires the use of both harsh conditions and strong acids or bases. For these reasons and to increase the value of the recycled product, improved recycling methods are needed. The molecular recycling of PET *via* depolymerisation into its building blocks (for subsequent assembly into regenerated PET) that can operate under milder reaction conditions is an attractive alternative, providing a strategy towards a more sustainable, circular economy.

**Scheme 1:**
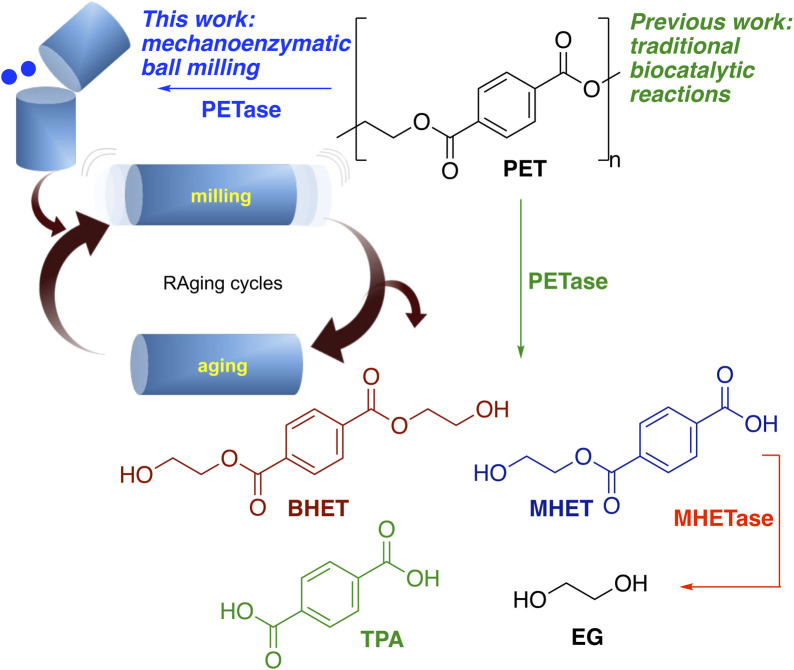
Products formed from the degradation of PET using PETases and previous work and the approach adopted here using mechanoenzymatic methods.

Although PET is particularly resistant to biodegradation,^3,4^ a number of naturally occurring PET hydrolases have been reported.^5–8^ Amongst these are cutinases from *Fusarium solani*,^9–12^ a leaf-branch metagenome (LCC)^6,13–15^ and *Humicola insolens* (HiC)^11,16,17^ as well as a hydrolase from *Thermobifida fusca* (TfH).^9,10,18,19^ In 2016, the first known bacterium capable of using PET as its sole carbon source, *Ideonella sakaiensis* (*Is*), was discovered.^15^ The authors identified a key enzyme (*Is*-PETase), responsible for the hydrolysis of PET into bis(2-hydroxyethyl) terephthalate (BHET), mono(2-hydroxyethyl) terephthalate (MHET), terephthalic acid (TPA) and ethylene glycol (EG). They also identified a secondary enzyme, MHETase, capable of degrading intermediate MHET into TPA and EG (Scheme 1). Recent interest has led to a surge in enzyme engineering studies yielding variants with improved activities and thermal stabilities.^20–28^ For example, a machine learning designed PETase with 4–5 fold higher activity than other engineered PET hydrolases has been reported.^27^ Untreated, post-consumer PET plastics were almost fully degraded by the mutant (FAST-PETase) within a week. Furthermore, they demonstrated a closed-loop recycling system by the recovery of monomers, highlighting the potential of degradative PETases in the molecular recycling of PET.

Mechanochemical reactions are gaining traction as a green method of performing organic synthesis, with a significant reduction in solvent usage. Recently, mechanochemistry has been adopted in the biocatalytic sphere giving rise to mechanoenzymatic reactions.^29–33^ A sustainable and efficient strategy for conducting various enzymatic transformations, the lack of bulk solvent avoids problems due to the insolubility of substrates, provides enzymes at high concentrations, and is said to more closely mimic the natural environment of *in vivo* enzyme reactions than traditional biocatalytic reactions in aqueous media.^34–39^ Specifically, the employment of liquid assisted grinding (LAG) conditions, where the ratio of the liquid volume to the mass of the solid reagents typically has an upper limit of 2.0 μL mg^−1^, is proposed to better mimic the native biological context in which microbe-secreted polymer degrading enzymes operate.^34,40^ Reports of efficient mechanoenzymatic transformations include: the degradation of poorly soluble natural polymers such as cellulose, lignocellulose and chitin^41,42^ by proteases^37^ and glycosyl hydrolases.^32,41–44^ Methodology coined reactive aging (RAging) has also been developed, where substrates and enzymes are subjected to repeated cycles of ball milling (reactive) followed by a period of incubation (aging), allowing for reactions to cool down to avoid enzyme denaturation.^41,42^

Recent studies have highlighted the mechanoenzymatic depolymerisation of PET using lyophilised HiC (Novozym^®^ 51032).^17,45^ In the HiC degradation of PET powder under ‘moist-solid’ conditions, short milling times were used followed by long periods of aging, leading to good yields of TPA.^17,45^

Immobilised enzymes and non-immobilised purified enzymes in solution or lysates are normally used for mechanoenzymatic reactions, for example, immobilised CALB has been described together with RAging procedures.^46,47^ In a further development, whole cell mechanoenzymatic strategies have been reported using transaminases under RAging conditions. Significantly increased yields of primary and secondary amines from aldehydes and ketones were generated compared to traditional solution reactions.^48^ The use of whole cells has the benefit of simplifying the enzyme preparation process, removing the need for cell lysis and enzyme purification. Action of the ball mill can effectively lyse the cells *in situ*, and in a low bulk solvent environment provides high enzyme concentrations, which together with assisted grinding can promote the reactions.^48,49^

In this study, for the first time, whole cell PETase mechanoenzymatic hydrolytic approaches and the effect of RAging on the breakdown of BHET and PET have been investigated (Scheme 1). This revealed significant advantages over traditional large bulk solvent based hydrolytic enzymatic reactions, with a marked increase in hydrolytic capability of a range of PET hydrolases. It is ideally suited to the molecular recycling of polymers such as PET, providing a proof-of-concept for future mechanoenzymatic depolymerisation reactions, where industrial process will necessarily use cell lysates due to the high costs of enzyme purification.

## Results and discussion

### Enzyme selection

Initially six PETases with potentially interesting properties in this system were selected (Table 1). These were, *Is*-PETase, *Polyangium brachysporum* PETase (*Pb*-PETase), *Acidovorax delafieldii* PETase (*Ad*-PETase), *Burkholderiales bacterium* PETase (*Bb*-PETase), and PET2 from an uncultured organism.^25,28^ For *Is*-PETase, the S238F/W159H double mutant was used which was shown to have an increased activity towards PET film and powder.^21,50^*Pb*-PETase and *Bb*-PETase have been used as purified enzymes for the degradation of PET.^5,51,52^ Sequence analysis showed that the protein sequence of *Bb*-PETase was longer on the N-terminus than other PETases (Fig. S1†) and a truncated enzyme has been reported.^52^ Here, 141 amino-acid residues were removed from the N-terminus, and the resulting subcloned gene named short *Bb*-PETase. *Ad*-PETase has been highlighted as a potential PETase, and is known to degrade poly(tetramethylene succinate), but to the best of our knowledge it has not been described to degrade PET and was therefore included.^25,53^ Finally, PET2 from an uncultured marine bacterium has been noted as thermophilic, which may be advantageous in ball mill experiments. It has previously been screened on agar plates against PET nanoparticles, as well as small scale PET-film degradation experiments.^5,28^

**Table 1:** PETases used in this study

Enzyme	Bacteria	UniProtKB/GenBank
*Is*-PETase S238F/W159H^21,50^	*Ideonella sakaiensis* 201-F6	A0A0K8P6T7
*Pb*-PETase^5,25,54^	*Polyangium brachysporum* DSM7029	A0A0G3BI90
*Bb*-PETase^25,51,52^	*Burkholderiales bacterium* RIFCSPLOWO2_02_FULL_57_36	OGB27210.1
Short*Bb*-PETase^52^	*Burkholderiales bacterium*	
*Ad*-PETase^25,53^	*Acidovorax delafieldii* BS-3	BAB86909.1
PET2 ^5,28^	Uncultured bacterium	C3RYL0

All PETases were ordered as synthetic genes, subcloned with the C-terminal His_6_-tag and expressed in the Shuffle T7 Express strain. PETases are typically used as purified enzyme preparations; there are many fewer examples where enzyme lysates are utilised.^27,55^ Here, with development in mechanoenzymatic reactions, all enzymes were first used as cell-free lysates.

### Initial small scale assays with BHET using enzyme lysates

Initial screens were carried out using BHET to first determine the relative hydrolytic activity of the PETases, using enzyme lysates. Different reaction temperatures were selected as it is widely reported that efficient PET hydrolysis occurs at higher temperatures,^56^ due to its high glass transition temperature (between 67 and 81 °C depending on crystallinity).^57^ Additionally, as PET chemical recycling methods include both acid and alkali hydrolysis,^58^ reactions were initially carried out at varying pHs (6.0–9.0). The full consumption of BHET was observed at 30 °C and pH 7.5 with four enzymes; *Is*-PETase S238F/W159H, *Pb*-PETase, short*Bb*-PETase and PET2 (Fig. S5†). Much lower conversions were observed for *Ad*-PETase and *Bb*-PETase, and both yielded only MHET. This likely reflects the propensity of *Ad*-PETase to degrade aliphatic polymers and *Bb*-PETase has been described to primarily degrade PET to MHET, rather than TPA.^52,53^ While the background hydrolysis of BHET increased between pH 6.0 and 9.0, generally no significant effect of pH on enzyme activity was observed. At 50 °C, *Ad*-PETase and *Bb*-PETase showed little if any activity above background levels, while for *Pb*-PETase and short*Bb*-PETase yields (by HPLC) dropped to 38% and 67% respectively. *Is*-PETase S238F/W159H and PET2, both showed full conversion at 50 °C, with a slight decrease in the yield of TPA for *Is*-PETase S238F/W159H. For thermophilic PET2,^5,28^ the yield of TPA and MHET increased with temperature. At 70 °C, BHET was fully hydrolysed in the absence of enzyme, due to non-enzymatic hydrolysis.

### Preliminary small scale PET assays

**Fig. 1:**
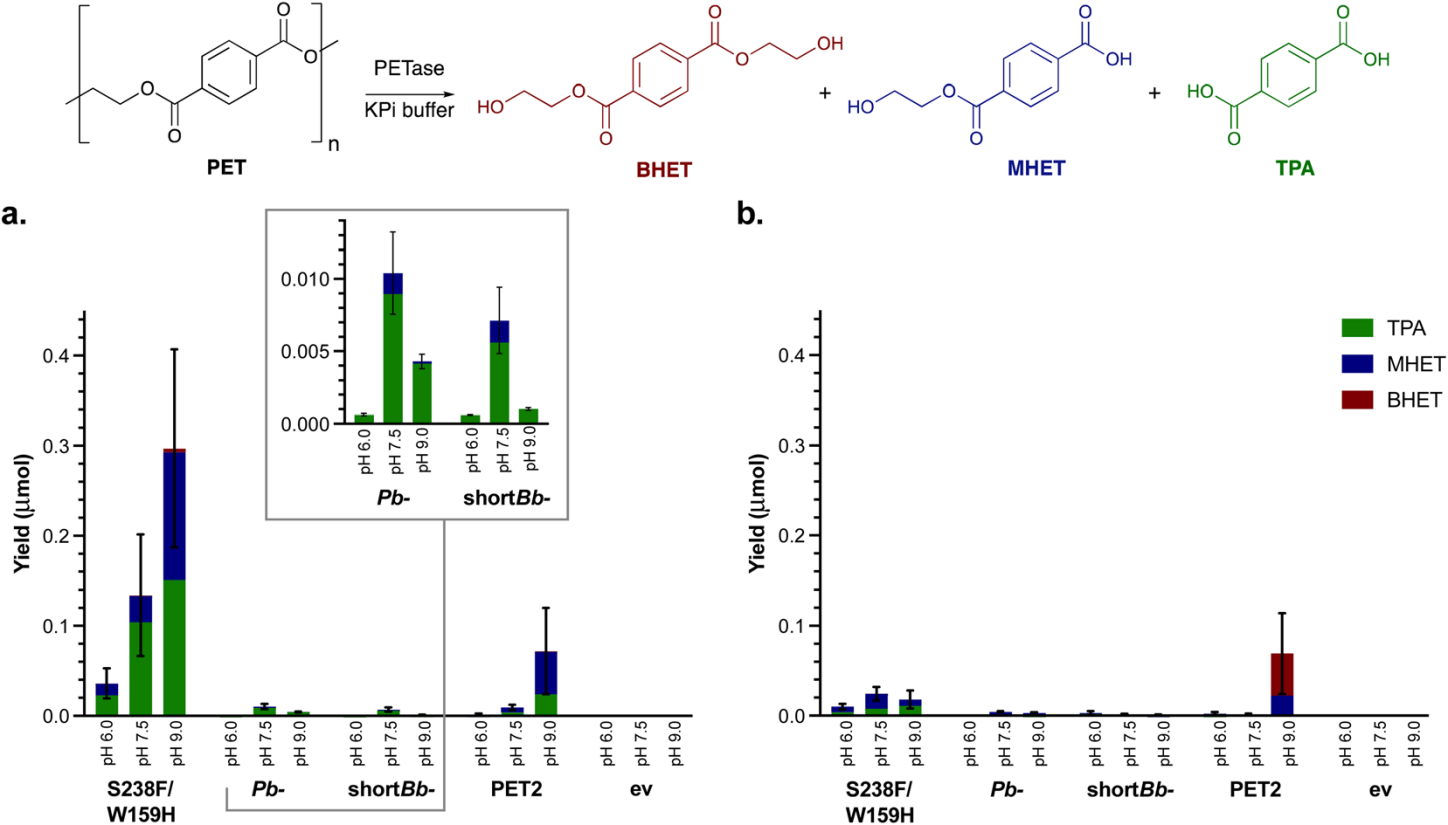
Small scale solution hydrolysis of PET powder. PET powder (4 mg mL^−1^), clarified lysate (1 mg mL^−1^) in KPi buffer (50 mM) for 96 h with shaking (700 rpm) testing the effect of (a) pH and (b) 10% DMSO on PET degradation by the four selected PETases. ev indicates the empty vector negative control.

Small scale assays were then carried out using the four most active PETases: *Is*-PETase S238F/W159H, *Pb*-PETase, short*Bb*-PETase and PET2. Total protein expression levels varied (0.16–0.45 mg protein per mg whole cell) with PETases produced at up to 18% total protein present (see ESI 1.3†). PET2 was expressed at notably lower levels. PET films are produced in a range of thicknesses and crystallinities leading to variable results, so commercial PET powder (Goodfellow, 300 microns, >48% crystallinity) was used to characterise the enzymes. Negative controls were carried out using a whole cell BL21 empty vector (ev) (comparison experiments with a whole cell SHuffle T7 empty vector strain also showed no degradation in solution). Reactions were completed in triplicate, at 1 mg mL^−1^ and 4 mg mL^−1^ concentrations of PET powder, and varying pHs to determine whether the degradation profile was similar to that with BHET. Initial reactions for 24 h showed little if any degradation. For this reason, and following literature precedent, reactions were carried out for 96 h at 30 °C (Fig. 1a and S6†).^15,50^*Is*-PETase S238F/W159H was shown to be the most active at pH 9.0, as was PET2.^5^ A pH of 7.5 proved to be best for *Pb*-PETase and short*Bb*-PETase which had similar activities. Interestingly little BHET was observed, with TPA being the major breakdown product in all cases at pH 7.5 (above 80% of the breakdown products were TPA), and a higher proportion of MHET was detected at pH 9.0. However, across all the enzymes, the most extensive degradation to TPA was observed at pH 7.5. Negative controls showed no degradation.

Recently, the addition of 10% DMSO to PETase reactions has been observed to increase the depolymerisation of amorphous PET film.^50^ Reactions were therefore also carried out with 10% DMSO (Fig. 1b). For PET2, *Pb*-PETase and short*Bb*-PETase, no difference in degradation activity was observed, possibly due to the higher crystallinity PET powder used here compared to amorphous PET film used in the literature. Additionally, S238F/W159H PETase activity appeared to be inhibited by 10% DMSO, further highlighting that DMSO was not beneficial.

### Mechanoenzymatic BHET breakdown

Mechanoenzymatic reactions with BHET using whole cell PETases and ball milling were explored in tandem with traditional biocatalytic solution reactions to better understand the effect of milling on the reaction.

This was attempted as enhancements have been seen in other hydrolytic reactions and more recently transaminase reactions with whole cells.^17,36–39,41–44,48^ It was also considered that lysing the cells *in situ* to release the enzymes *via* mechanical milling might be beneficial.^48^ For polymer-assisted grinding it has been stated that having more matter inside the jars can promote reactions, so inert additives in the form of cell debris might also aid the reaction.^43^ RAging cycles were employed to mitigate against enzyme denaturation due to increased temperatures from mechanical forces.^41,42,44^

**Fig. 2:**
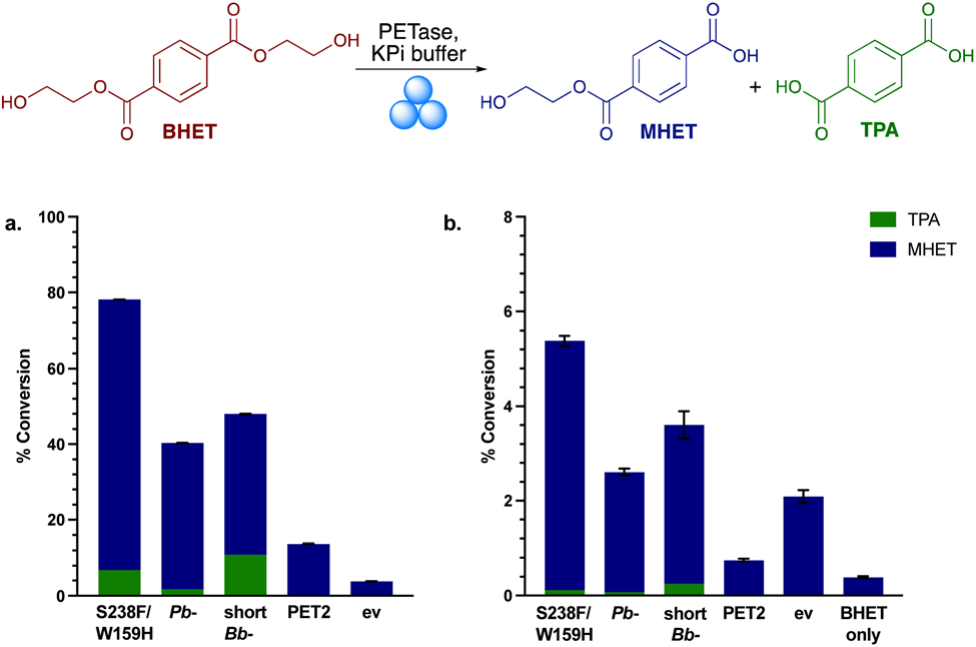
Mechanoenzymatic BHET hydrolysis with the PETases. (a) Conversion yields of MHET and TPA with lyophilised whole cell PETase (50 mg) and BHET powder (200 mg) in KPi buffer. (b) Conversion yield of MHET and TPA in the mechanoenzymatic breakdown of BHET. RAging cycles consisted of 20 min milling, 40 min aging × 8 cycles at 30 Hz. ev indicates the empty vector negative control using whole cell BL21 ev.

The four PETases were used as lyophilised whole cells with BHET in solution reactions at 30 °C and pH 7.5. In agreement with earlier results, the most active PETase was *Is*-PETase S238F/W159H, with nearly 80% yields (by HPLC) after 8 h (Fig. 2a). Interestingly, *Pb*-PETase exhibited slightly lower activity than short*Bb*-PETase. The proportion of TPA generated by short*Bb*-PETase was higher than with *Pb*-PETase, consistent with the small scale lysate reactions with BHET (Fig. S5†). PET2 exhibited low levels of degradation. With all enzymes, MHET was the predominant breakdown product, and the levels of degradation were promising. In the environment, PETases are secreted where they degrade PET extracellularly. It is likely that BHET, as a small molecule, can be taken up by the PETase secreting cells, resulting in hydrolysis in the absence of cell lysis. Little degradation was observed in the negative control.

Mechanoenzymatic reactions were then carried out with 8 RAging cycles of 20 min milling supplemented by 40 min aging, under LAG conditions (Fig. 2b). Again, *Is*-PETase S238F/W159H exhibited the highest amount of BHET degradation, followed by short*Bb*-PETase, *Pb*-PETase then PET2, with a similar proportion of degradation to TPA observed across S238F/W159H, *Pb*- and short*Bb*-PETases, as in solution. The negative control, ev, exhibited higher degradation levels than PET2, suggesting a mechanochemical effect. To explore the particulate-grinding effect on BHET it was subjected to the milling process on its own. Low levels of hydrolysis were seen, with some MHET formed. Overall, the low levels of BHET breakdown in the mechanoenzymatic reactions compared to in solution, was rationalised as probably due to the enzymes being denatured.

### Mechanoenzymatic PETase reactions

**Fig. 3:**
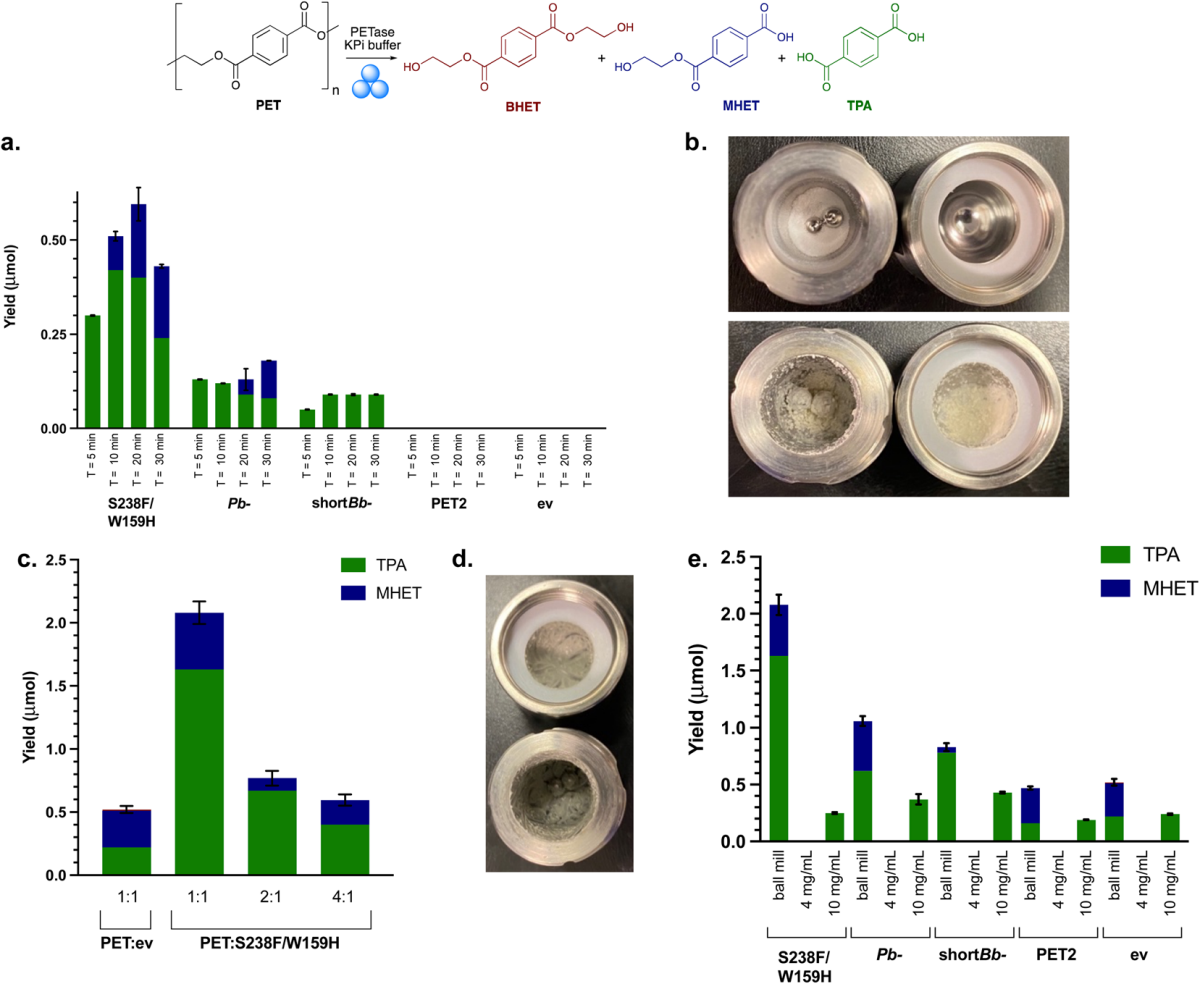
Initial mechanoenzymatic PETase reactions. (a) Ball milling yields after 8 h, with RAging cycles of *T* = 5, 10, 20 or 30 min milling at 30 Hz, followed by 55, 50, 40, 30 min aging, respectively. Reaction conditions: PET powder (200 mg), lyophilised whole cell PETase (50 mg) and KPi buffer (200 μL, pH 7.5). (b) Images depicting PET powder in ball milling jars before (top) and after (bottom) the reactions. (c) Ball milling yields after varying the ratio (1 : 1, 2 : 1, 4 : 1) of PET powder (50 mg) to *Is*-PETase S238F/W159H using optimal RAging cycles of *T* = 20 min milling and 40 min aging × 8 cycles, at 30 Hz. (d) Differing consistency of the reaction mixture post 8 h cycle with the LAG value increased from 1.5 μL mg^−1^ to 2.0 μL mg^−1^. (e) Ball milling compared with solution reactions with PET powder substrate (50 mg) and lyophilised whole cell *Is*-PETase S238F/W159H (50 mg), 8 h total reaction time. ev indicates the empty vector negative control using whole cell BL21 ev. Experiments with a whole cell SHuffle T7 empty vector strain also showed comparable degradation under mechanoenzymatic conditions (Fig. S7†). Also, 4 mg mL^−1^ and 10 mg mL^−1^ refers to the concentration of the PET powder starting material.

PET degradation reactions were then carried out using PET powder under mechanoenzymatic conditions. Hammerer *et al.* have recently reported increased yields of enzymatic hydrolysis of microcrystalline cellulose with RAging cycles of 5 min milling followed by 55 min aging.^41^ Similarly, recent reports of mechanoenzymatic PET degradation by HiC also employed a short RAging cycle of 5 min milling, 7 days aging.^17,45^ A key difference here is our use of whole cell enzymes, and it was considered that more RAging cycles would be necessary to lyse the cells *in situ*. To therefore assess the best RAging cycle for the mechanoenzymatic hydrolysis of PET, reactions comprising 200 mg PET powder and 50 mg lyophilised whole cell PETase were subjected to RAging cycles of either 5, 10, 20 or 30 min milling followed by 55, 50, 40 or 30 min aging, respectively (Fig. 3a). After 8 h of RAging (*i.e.* 8 × the above cycles) the products appeared as a paste (Fig. 3b). HPLC analysis indicated the release of degradation products when subjected to RAging cycles with all enzymes, other than PET2. Interestingly, TPA was the major product for all PETases, accounting for 100% of the product with active enzymes using 5 min milling, and across all the milling times with short*Bb*-PETase. *Is*-PETase S238F/W159H exhibited the highest level of degradation, consistent with previous reactions, with 20 min milling and 40 min aging giving the highest yields. Similar levels of TPA were observed with the 10 and 20 min milling RAging cycles, but increased levels of MHET were detected with 20 min. It was thought that the decrease in yield for *Is*-PETase S238F/W159H with 30 min milling may be due to the enzyme denaturing. *Pb*-PETase showed similar levels of degradation across each RAging cycle, with a slightly higher proportion of MHET formed after 20 and 30 min milling cycles.

PET2 with PET powder showed no degradation. Mechanoenzymatic reactions were therefore repeated with a longer reaction time of 24 h, using both purified and lysate PET2, as degradation had been previously observed with lysate in the small scale assay (4 mg mL^−1^ PET powder and 1 mg mL^−1^ PETase after 96 h). Activity was confirmed with good levels of breakdown products observed (Fig. S8†) at lower substrate loadings (50 mg PET) with purified enzyme. At higher substrate loadings (200 mg) and clarified lysate (from 50 mg lyophilised whole cells), only trace amounts of MHET and TPA were formed. Clearly the relatively low enzyme expression of PET2 (ESI 1.3 and Fig. S2†) had an impact on the level of degradation and substrate loading is also important.

### Investigations into substrate–enzyme loading

To establish the optimal ratio of whole cell PETases used to the amount of PET substrate used, the quantity of PETase whole cells was increased from 50 mg to 200 mg (1 : 1 w/w), and subjected to mechanoenzymatic treatment, with a *T* = 20 min milling time selected as it had previously given good results. However, product analysis proved problematic, as the mixture formed was difficult to solubilise. Therefore, to balance the amount of solid material in the milling jars, the quantity of PET powder was decreased to 100 mg and 50 mg (2 : 1 w/w and 1 : 1 w/w, PET powder : PETase). This still adhered to LAG conditions, with the value of *η* equalling the upper limit of 2.0 μL mg^−1^, rather than 1.5 μL mg^−1^. The yield of breakdown products increased nearly 4-fold when compared with using 200 mg PET powder starting material (Fig. 3c). This did not arise from increased background degradation due to the action of the milling (see negative control, ev).

### PET degradation using PETases in solution

To highlight the effect of using the ball mill over traditional biocatalytic reactions, as well as carrying out solution reactions with whole cells for direct comparison with mechanoenzymatic reactions, solution reactions with freshly prepared cell lysates were performed. Comparability between the amount of enzyme in each of the solution reactions was ensured by resuspending and lysing 50 mg of whole cells. Cell lysate is often used in biocatalytic reactions, avoiding the protein purification step, and saving on labour, cost and time. Both whole cell and lysate solution reactions with 50 mg PET powder (4 mg mL^−1^ substrate concentration), based on the small scale assays, yielded no degradation, highlighting the positive effect of the ball mill and whole cell enzymes in the mechanoenzymatic system. The concentration of PET was increased to 10 mg mL^−1^ (along with increasing the enzyme concentration) by scaling down the volume of buffer to highlight the hydrolytic capability of PETase lysates in traditional biocatalytic solution reactions (Fig. 3e).

The lack of degradation for solution reactions compared to the successful mechanoenzymatic PET breakdown observed, suggests that the ball milling is an essential feature here for the hydrolysis of PET. Combined with the lower levels of breakdown observed in the mechanoenzymatic negative controls, this strongly suggests that an additive effect is observed between the PETase and the ball milling technique which causes PET degradation.

### Other PET materials

**Fig. 4:**
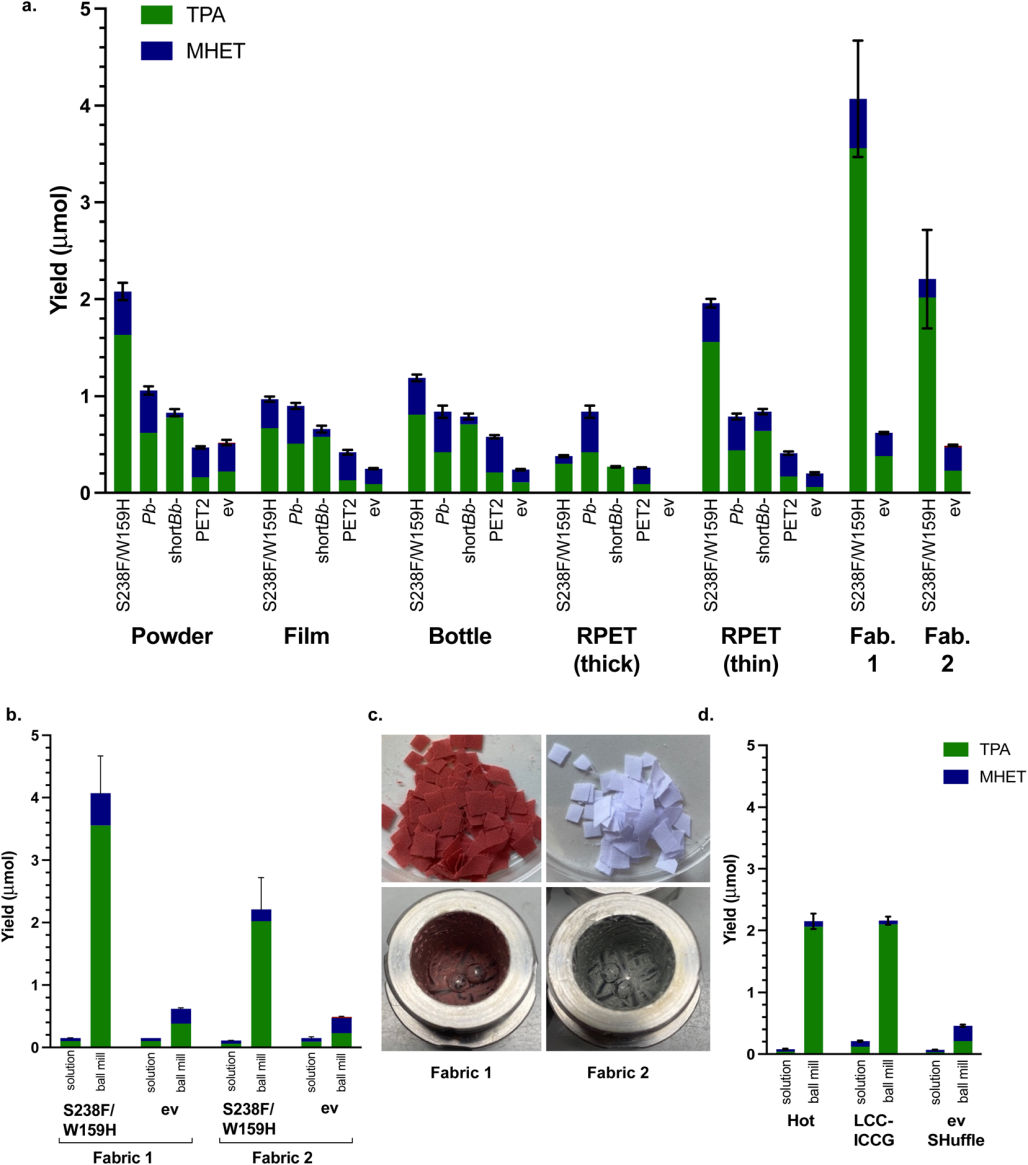
The use of different PET materials. (a) PETases with PET powder, films and bottles using optimised mechanoenzymatic conditions of *T* = 20 min milling, 40 min aging × 8 cycles, at 30 Hz, substrate (50 mg), lyophilised whole cell PETase (50 mg), KPi buffer (200 μL, pH 7.5) and negative control (ev). (b) Comparison between mechanoenzymatic ball milling degradation and solution degradation of fabric 1 and fabric 2 and solution reactions on a 5 mL scale. (c) Images of RPET fabric 1 and fabric 2 before and after mechanoenzymatic degradation. (d) Mechanoenzymatic degradation compared with solution degradation (5 mL total reaction volume) of PET powder using optimised conditions and whole cell literature PET hydrolytic enzymes HotPETase^55^ and LCC-ICCG,^6^ compared with a SHuffle ev control.

In order to show the feasibility of utilising this method more generally, other forms of PET were tested. There are far fewer reports in the literature of using PET powder to date, most experimentation thus far has used PET film. A sample of PET film (Goodfellow, 0.25 mm thickness, 40–60% crystallinity) was therefore used to compare with PET powder, as well as a post-consumer PET drinks bottle sample (0.15 mm thickness) and some recycled PET (RPET (thick) 0.30 mm thickness) from a post-consumer yoghurt pot. The three films were prepared (6 mm discs), washed with soapy water, water and ethanol, then dried overnight. The conditions used were *T* = 20 min milling (40 min aging, 8 cycles), 50 mg substrate and 50 mg whole cell PETase. Again, *Is*-PETase S238F/W159H exhibited the highest degradation across all the materials, except for RPET (thick) which was degraded most fully by *Pb*-PETase (Fig. 4a). Indeed, only partially broken-down discs were isolated after 8 h, in contrast to the other PET films which formed a smooth paste with no film remaining. RPET may contain other contaminants and it may be possible that *Pb*-PETase can tolerate such impurities more readily. The thickness of the material will also be an important factor.

To explore this, a thinner post-consumer food packaging RPET sample (RPET (thin) 0.19 mm thickness) was tested and *Is*-PETase S238F/W159H then exhibited similar levels of degradation to PET powder. PET powder was more readily degraded than most of the films (only RPET (thin) was similar), which may have been due to the smaller particle size, despite the higher crystallinity (in comparison to the Goodfellow PET film). The enzyme showing the most consistent tolerance towards the PET materials and proportionately fullest degradation to TPA was short *Bb*-PETase, reflecting the previous experiments with BHET and PET. Overall, these results highlighted a good performance for the PETases under mechanoenzymatic conditions with a range of PET materials. Solution reactions with whole cells were carried out for comparison purposes, and in all cases no degradation was detected.

Carrying forward the optimised conditions and using the best performing *Is*-PETase S238F/W159H, two samples of RPET fabric (Table 2) were also subjected to mechanoenzymatic degradation (Fig. 4b and c). Fabric 1 was degraded more readily than all of the materials tested, with a with a yield of over 4.0 μmol and a 6.5-fold increase compared with the ev control. Similarly, fabric 2 was also well degraded with a combined TPA and MHET yield of over 2.20 μmol. In comparison with the equivalent solution lysate reactions, carried out at 10 mg mL^−1^ based on previous PET powder solution reactions, the mechanoenzymatic process gave a 27-fold increase in yield for the degradation of fabric 1 and a 20-fold increase for the degradation of fabric 2 (Fig. 4b). The appearance of the fabrics before and after degradation is shown in Fig. 4c.

**Table 2:** Fabric information on the PET fabrics used

Fabric	Composition	Weight (g m^−2^)	Thickness (mm)	Finishing	Source
1	100% RPET	120	0.15	None	Waste2Wear
2	65% RPET, 35% cotton	115	0.17	Teflon^®^	Waste2Wear

### Other recent PET hydrolases

Based on the efficiency of recent literature PET hydrolases that can effectively break down amorphous and semi-crystalline PET substrates when used as purified enzyme, a couple were selected for further mechanoenzymatic reactions using the reaction conditions developed. Interestingly, while the overall combined yields of TPA and MHET did not increase, a significant increase in degradation between comparable solution reactions and mechanoenzymatic reactions against the SHuffle ev control was again observed, with a 26-fold increase for HotPETase^55^ and a 10-fold increase for LCC-ICCG^6^ (Fig. 4d). This again highlighted the advantages of applying the PETases mechanoenzymatically.

### To buffer or not

As a final note, the small scale PET powder assays highlighted that for both *Is*-PETase S238F/W159H and PET2, pH 9.0 buffer enabled a higher degradation of PET. To see if this influenced the mechanoenzymatic reaction, pH 9.0 buffer was used. For both *Is*-PETase S238F/W159H and PET2, a 15% and 20% increase in yield was observed, respectively (Fig. S10†). To demonstrate that the observed increase was not purely a result of the higher basicity of the KPi buffer, a negative control with whole cell ev was performed. For the pH 7.5 and pH 9.0 controls, there was a decrease in yield of just over 5%, confirming a pH effect with the PETase. Further to this, PET powder breakdown using *Is*-PETase S238F/W159H was explored replacing KPi buffer with deionised water, providing comparable levels of degradation to the pH 7.5 reaction. This highlighted another advantage of the system developed, removing the need for buffers, which could potentially aid the green chemistry profile.

## Conclusions

In summary, we report the first use of whole cell PETase enzymes to breakdown a variety of PET materials (including post-consumer samples) and BHET using mechanoenzymatic ball milling. The activity of a short*Bb*-PETase was also explored with several forms of PET and highlighted that a more complete conversion to TPA was possible than the other PETases explored (consistently above 75% of the total product formed). An additive effect between the ball milling technique and PETase action was demonstrated to breakdown PET where comparable traditional biocatalytic reactions in aqueous solutions with whole cell enzymes or lysate were significantly less effective. This simplification in the preparation of the enzyme is notable, particularly as most reported PETases to date are used as purified enzymes. While the use of whole cells, due to the lower amounts of enzymes, results in lower TPA yields compared to literature reports, it highlights a valuable approach which can be applied on larger scales where the costs of enzyme purification are prohibitive.

Benefits of the mechanoenzymatic breakdown of PET include: a significant reduction in solvent usage, approximately 30-fold when compared with the leading large scale industrial PET breakdown system^6^ and 2600-fold when compared with the current leading PETase hydrolysis of PET^27^ (56 μL solvent per nmol and 5 mL solvent per nmol *versus* 1.9 μL per nmol in this work). Some ball milling is already used in industrial settings to breakdown PET for secondary recycling, enhancing the feasibility of upscaling this process with PETases in the future. Yields of breakdown products were increased by implementation of a 1 : 1 w/w substrate to whole cell PETase ratio, under LAG conditions, even in water. We are now exploring this methodology with other PETases and tackling PETase expression levels. It is hoped that in the future mechanoenzymatic processes could offer a solution to the single-use plastic waste crisis and aid the transition towards a circular economy.

## Author contributions

E. A.-D., L. L., and E. M. C. investigated the mechanochemical enzymatic reactions and developed the degradation methodologies. M. B., D. D., G. S., and J. J. developed the enzymes. The manuscript original-draft was written by E. A.-D., L. L., M. B., D. D., and H. C. H. The project conceptualisation was by all authors and supervised by T. D. S., J. W. E. J., J. M. W., and H. C. H. The manuscript has been reviewed and edited by all contributing authors.

## Conflicts of interest

There are no conflicts to declare.

## Note added after first publication

This article replaces the version published on 29 March 2023, which contained an incomplete version of Scheme 1.

## Supplementary Material

RA-013-D3RA01708G-s001
