# Supplementary material for: Mechanoenzymatic reactions for the hydrolysis of PET
Source: RSC Adv. 2023 Mar 29;13(15):9954–62. doi: 10.1039/d3ra01708g (PMC10050947; doi:10.1039/d3ra01708g)
Supplement: RA-013-D3RA01708G-s001 [file RA-013-D3RA01708G-s001.pdf]

Electronic Supplementary Material (ESI) for RSC Advances.
This journal is © The Royal Society of Chemistry 2023

Supplementary Information

Mechanoenzymatic Reactions for the Hydrolysis of PET

1. Chemical Biology

1.1 General methods

The following centrifuges were used: Beckman Avanti JXN-26 Series, Eppendorf Centrifuge 5415R, Eppendorf Centrifuge 5810R, Eppendorf Centrifuge 5430R. A Kuhner ShakerX ClimoShaker ISFI-X, New Brunswick Scientific Innova 44 or BIOER Mixing Block MB-102 incubating shaker was used. The autoclaves used were a Priorclave TACTROL 2 and a Priorclave TACTROL 3. Where sterilisation of waste and media was performed, the temperature was held at 121 °C for 30 min. Sonication of *E. coli* cells was performed using a Branson Sonifier 150 with microprobe tip at a power of 10 µ. Sonication of reaction mixtures for analysis was performed using a VWR Ultrasonic Cleaner USC-THD. Potassium phosphate buffer (50 mM, pH 7.5) was prepared from K_2HPO_4 and KH_2PO_4 according to the desired pH and made up to the total volume with distilled water.

1.2 Gene identification and cloning

For all proteins, the signal peptides were predicted by SignalP and then removed. All corresponding genes were codon optimized for expression in *E. coli* and ordered as synthetic genes (Eurofins Genomics) flanked with *SapI* or *BsaI* restriction sites. The synthetic genes were then subcloned into customized pET29a(+) with a C-terminal His_6-tag.[1]

```
Bb-PETase          1 MQVVVFEETFSTGLGKFTAAGSVVTSSGAARLDGCYGCTDGSITSTAIST     50

shortBb-PETase     1 --------------------------------------------------      0

Bb-PETase         51 VDFTGLRLSFDRVTSGLDSGEAGIAEFSTNGSTYTAVESIRTASGRVTFN    100

shortBb-PETase     1 --------------------------------------------------      0

Bb-PETase        101 LPTSAENQSGLRLRFRINASLSSETYTVDNIRLEGTSGSGGGTTNPFEKG    150
                                                              ..|||||||
shortBb-PETase     1 -----------------------------------------MQTNPFEKG      9

Bb-PETase        151 PDPTKTMLEASTGPFTYTTTTVSSTTASGYRQGTIYHPTNVTGPFAAVAV    200
                     ||||||||||||||||||||||||||||||||||||||||||||||||||
shortBb-PETase    10 PDPTKTMLEASTGPFTYTTTTVSSTTASGYRQGTIYHPTNVTGPFAAVAV     59

Bb-PETase        201 VPGYLASQSSINWWGPRLASHGFVVITIDTNSTSDQPPSRATQLMAALNQ    250
                     ||||||||||||||||||||||||||||||||||||||||||||||||||
shortBb-PETase    60 VPGYLASQSSINWWGPRLASHGFVVITIDTNSTSDQPPSRATQLMAALNQ    109

Bb-PETase        251 LKTFSNTSSHPIYRKVDPNRLGVMGWSMGGGGTLIAARDNPTLKAAIPFA    300
                     ||||||||||||||||||||||||||||||||||||||||||||||||||
shortBb-PETase   110 LKTFSNTSSHPIYRKVDPNRLGVMGWSMGGGGTLIAARDNPTLKAAIPFA    159

Bb-PETase        301 PWNSSTNFSTVSVPTLIIACESDSTAPVNSHASPFYNSLPSTTKKAYLEM    350
                     ||||||||||||||||||||||||||||||||||||||||||||||||||
shortBb-PETase   160 PWNSSTNFSTVSVPTLIIACESDSTAPVNSHASPFYNSLPSTTKKAYLEM    209

Bb-PETase        351 NNGSHSCANSGNSNAGLIGKYGVSWMKRFMDNDTRFSPYLCGAPHQADLS    400
                     ||||||||||||||||||||||||||||||||||||||||||||||||||
shortBb-PETase   210 NNGSHSCANSGNSNAGLIGKYGVSWMKRFMDNDTRFSPYLCGAPHQADLS    259
```

```
Bb-PETase          401 LTAIDEYRENCPY    413
                       |||||||||||||
shortBb-PETase     260 LTAIDEYRENCPY    272
```

Figure S1. Pairwise protein sequence alignment of *Bb*-PETase and short*Bb*-PETase. Signal peptides have been removed. Amino acids in grey are not present in short*Bb*-PETase.[1,2]

For the recent PET hydrolases characterised in the literature (labelled as HotPETase[3] and LCC-ICCG[4]), the codon-optimised genes were ordered as synthetic genes already in pET29a(+) with a C-terminal His_6-tag (Genscript). For expression, all plasmids were transformed into *E. coli* expression strain Shuffle T7 Express (NEB).

1.3 Protein expression and purification

E. coli Shuffle T7 Express cells (NEB) was used for protein expression with the following conditions. Overnight culture was grown in LB at 30 °C. The following morning it was diluted 1:100 in Magic Media (200 ml culture, 1L flask) and left shaking at 30 °C and 200 rpm for 6-7 h, then at 25 °C for 16 h. Cells were harvested by centrifugation and stored at -80 °C. The cell pellets were resuspended in KPi buffer (10 mL, 50 mM, pH 7.5) and freeze dried, stored at -20 °C and used as "whole cells" as needed. To prepare the clarified cell lysate, lyophilised cells (25 mg) were resuspended in KPi buffer (1 mL, 50 mM, pH 7.5) and disrupted by sonication (5 × 10 sec on, 10 sec off, 10 μ) and the cell lysate isolated *via* centrifugation (13, 500 rpm, 10 min, 4 °C). Clarified cell lysate was prepared fresh for each use and kept on ice at all times. The proteins were analysed by SDS-page (Figure S2). Total protein concentration was determined using a standard Bradford assay. Total protein concentrations given below are per mg of whole cell: *Is*-PETase S238F/W159H (0.33 mg/mg); *Pb*-PETase (0.45 mg/mg); short*Bb*-PETase (0.42 mg/mg); PET2 (0.16 mg/mg); HotPETase[3] (0.17 mg/mg); LCC-ICCG[4] (0.14 mg/mg). The proteins were purified using nickel-ion affinity using nickel-NTA agarose and analysed by SDS-page (Figure S2). The protein concentration present in the cell lysate was determined using ImageJ software as: *Is*-PETase S238F/W159H (18%); *Pb*-PETase (17%); short*Bb*-PETase (4%); PET2, HotPETase and LCC-ICCG (overexpression not distinguishable).

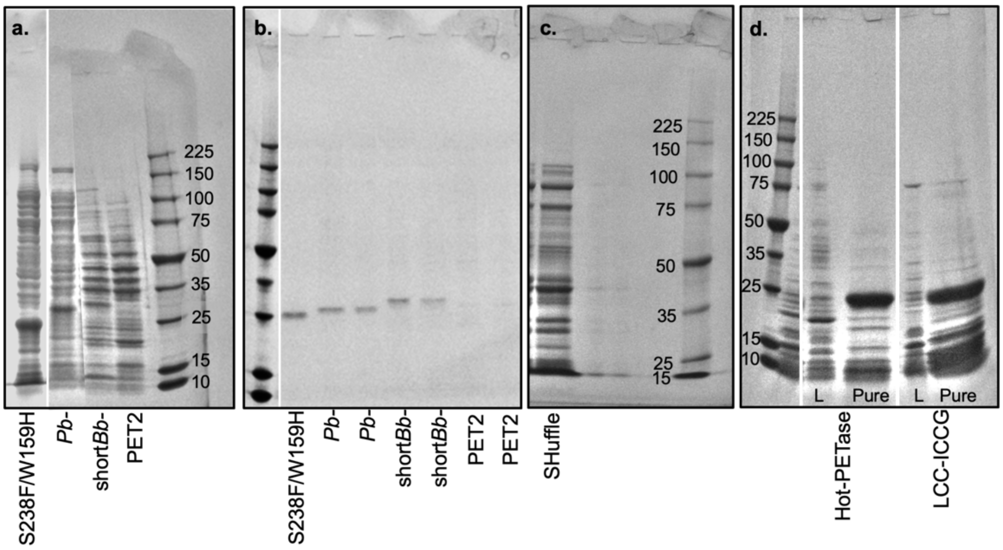

Figure S2. a. SDS-page gel showing expression of PETases in clarified lysate as labelled. **b.** Purified PETases shown as labelled. **c.** SHuffle T7 Express strain empty vector. **d.** Recent literature PETases grown and expressed, then purified using short Ni-NTA spin columns, L = lysate, 'Pure' = semi-purified. Broad range protein molecular weight marker 10-225 kDa. Black boxes indicate lanes from the same gel, where lanes have been cut out, a white border is shown. See Figure S2A below for original gels.

Figure S2A.
SDS-page original gels

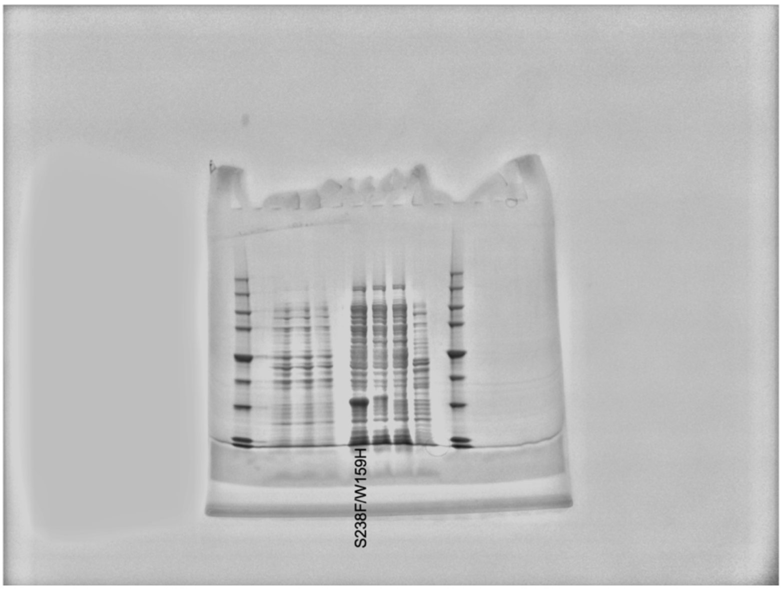

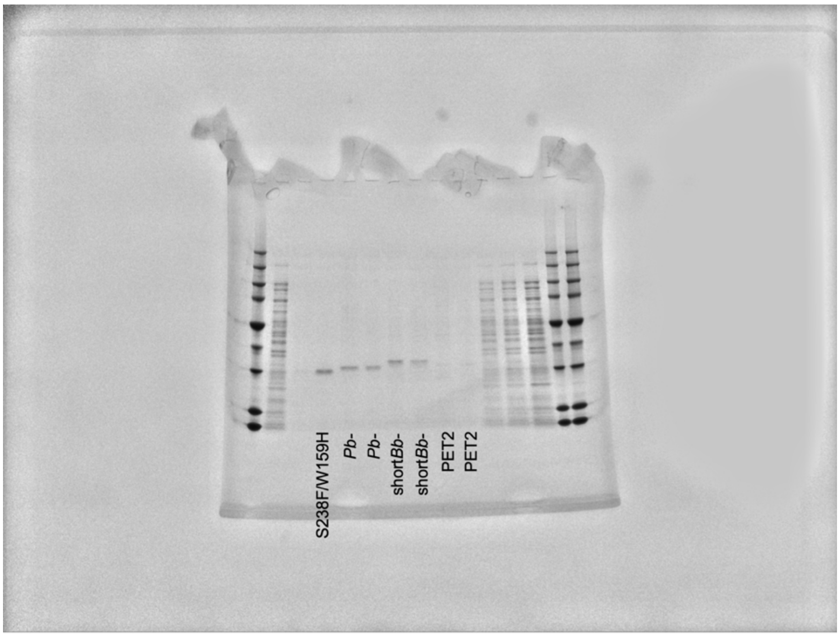
S238F/W159H
Pb-
Pb-
shortBb-
shortBb-
PET2
PET2

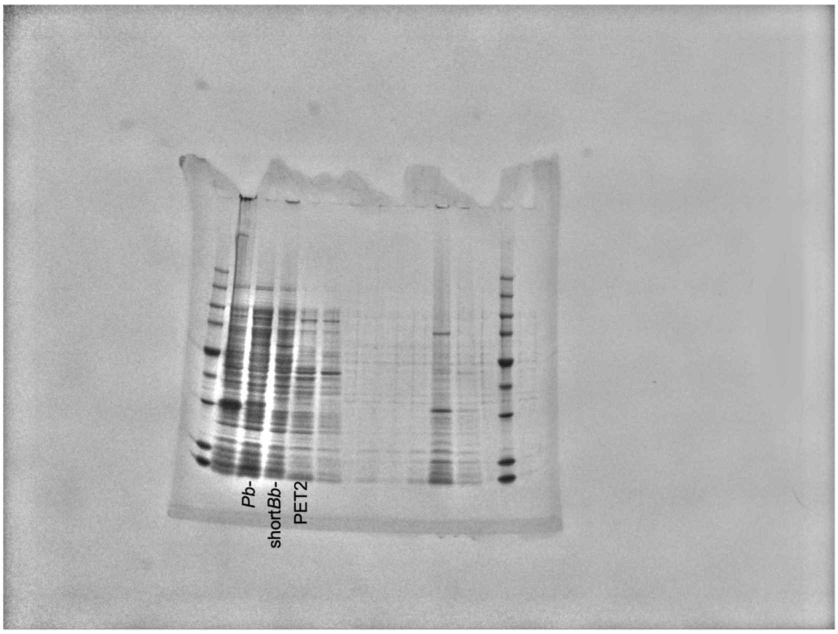
Pb-
shortBb-
PET2

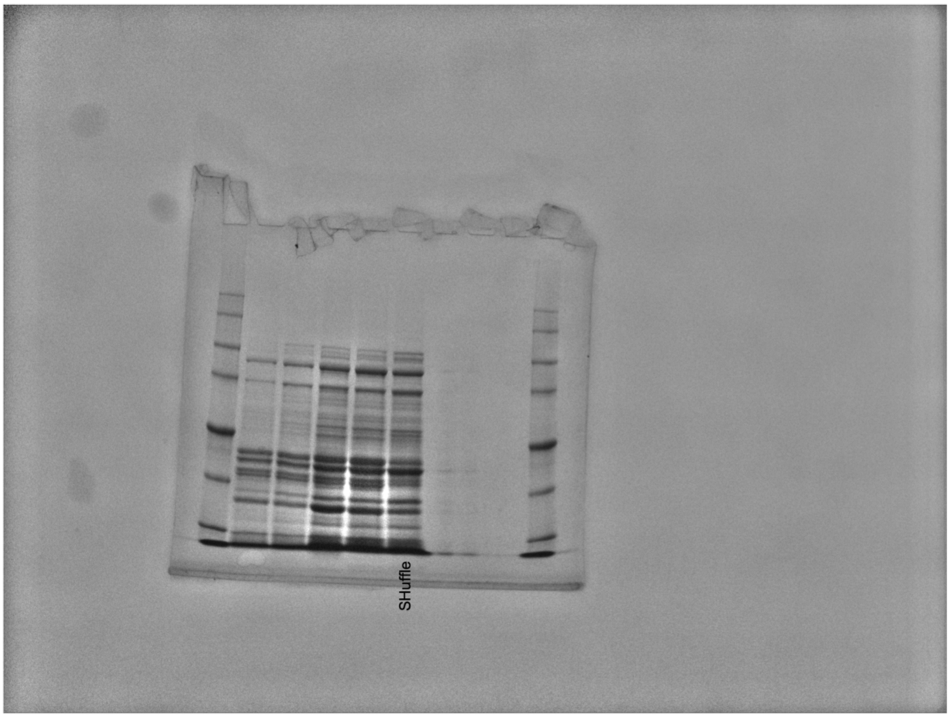
SHuffle

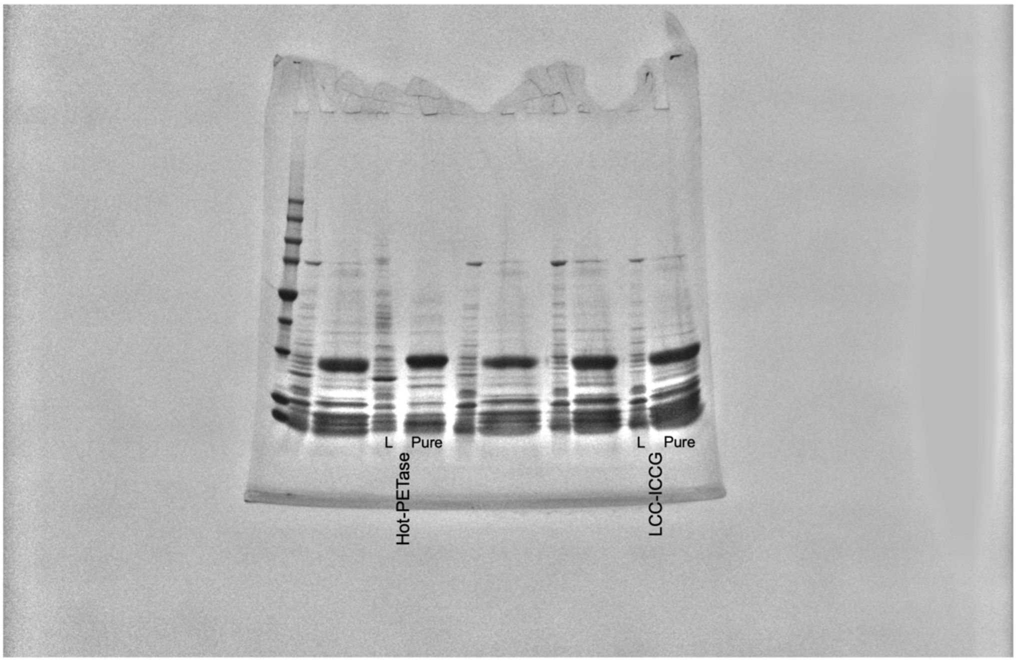
L
Pure
Hot-PETase
L
Pure
LCC-ICCG

1.4 Reverse phase analytical HPLC

HPLC was performed using an Agilent 1260 Infinity liquid chromatography system comprising of a G1329B autosampler, G1311C quaternary pump, 1260 G1316A column oven and 1260 G1314F variable wavelength detector. The system was equipped with a HiChrom ACE C18-AR column (250 mm x 4.6 mm). Acetonitrile (MeCN) in water (0.1% v/v TFA) was used as the mobile phase. The gradient used was 20% MeCN for 5.10 min, a linear gradient to 100% MeCN over 2.20 min, 100% MeCN for 1.30 min, a linear gradient to 20% MeCN over 20 s, followed by 20% MeCN for 4.40 min. A flow rate of 1 mL min^{-1} was used and injected samples were of 10 µL volume.

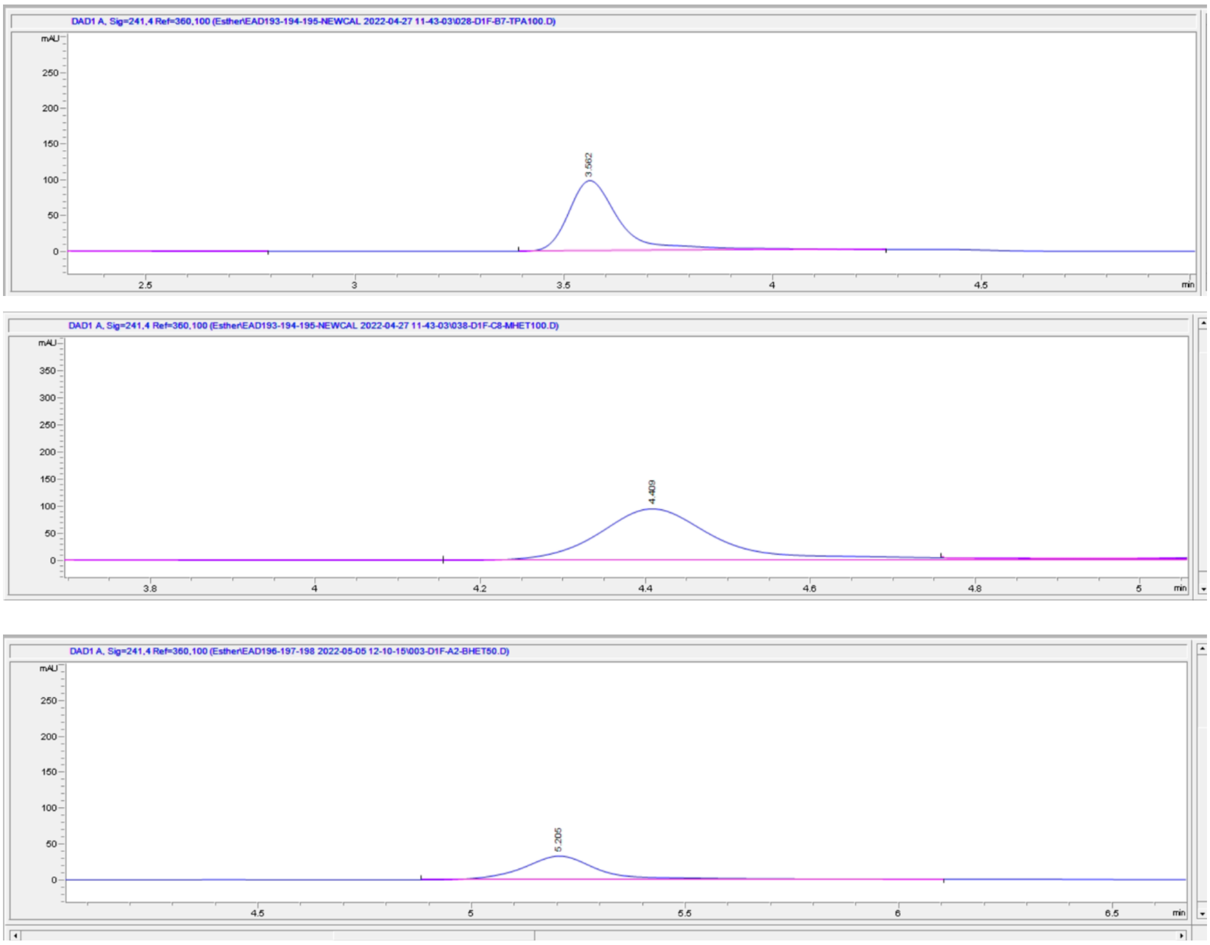

Figure S3. HPLC chromatograms showing retention times for TPA (3.56 min), MHET (4.41 min), BHET (5.21 min) at a detection wavelength of 241 nm.

1.5 Materials

All chemicals were obtained from chemical suppliers and were used without further purification. TPA and BHET were purchase from Sigma Aldrich. MHET was synthesised as indicated below. PET powder (300 microns, >48% crystallinity) and Biaxially oriented PET film (0.25 mm thickness, 40-60% crystallinity) were purchased from Goodfellow. A post-consumer PET bottle was used (PET bottle, 0.15 mm thickness) a post-consumer yoghurt pot made of RPET (RPET (thick), 0.30 mm thickness) and a post-consumer dessert packaging made of RPET (RPET (thin), 0.19 mm thickness) were used. No data is available for these but they were subjected to the same washing procedure as the commercial PET film. PET substrates were prepared by using a hole punch to cut discs of equal diameter, washed in warm soapy water, then thoroughly

rinsed with 100% EtOH and left to dry overnight. PET fabric samples were kindly provided by Waste2Wear: Fabric 1 was 100% RPET with construction 75Dx75D and Weight 120 g/sq. m, 0.15 mm thickness; Fabric 2 was 65% RPET 35% cotton with construction 45x45/144x70, weight 115 g/sq. m and an easy care/Teflon® finishing, 0.17 mm thickness.[4] PET fabrics were cut into squares of 5 mm, then washed thoroughly with 100% EtOH before being left to dry overnight.

1.6 Solubility test of BHET and TPA mixture

Stock solutions (10 mM) of BHET and TPA were made up in solvent systems to test the effect of the solvent system on the breakdown product solubility (Figure S4). Solvent systems were: **A** 50:50, DMSO:H_2O; **B** 70:20:10, H_2O:MeCN:formic acid; **C** 50:50, DMSO:50 mM KPi buffer pH 7.5; **D** MeCN; **E** 0.1 mM NaOH pH 9.0; **F** 0.01 mM NaOH pH 8.0; **G** 10 mM K_2HPO_4 pH 9.0; **H** 50:50, 0.01 mM NaOH:MeCN; **I** 50:50, 10 mM K_2HPO_4:MeCN; **J** 50:50, DMSO:H_2O (centrifuged); **K** 50:50, DMSO:H_2O (filtered). Samples were diluted by a factor of 20 (0.5 mM) with the respective solvent system and the solubility of BHET and TPA were analysed by analytical HPLC.

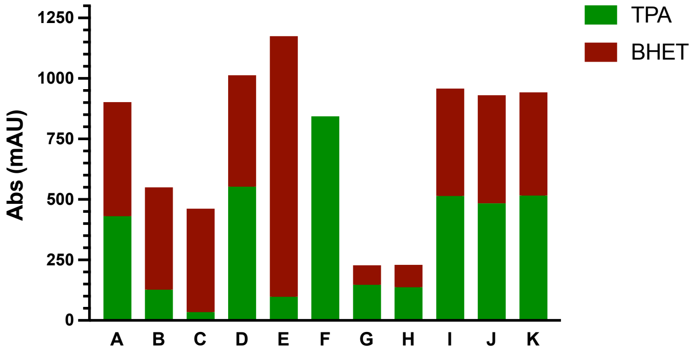

Figure S4. TPA and BHET mixture solubility test.

1.7 Assays and mechanoenzymatic reactions

Ball milling reactions were carried out using 10 mL stainless steel jars from Retsch, 2 x 5 mm stainless steel balls, and a Retsch MM500 vario ball mill. Negative controls were carried out using an empty vector (ev) of BL21 whole cell, grown in lysogeny broth.

BHET degradation assay. Enzymatic reactions were carried out in 96-well plates (200 µL system) containing clarified cell lysate (0.7-1.1 mg/mL), KPi buffer (50 mM, pH 7.5) and BHET (5 mM) with 20% DMSO to aid solubility. Reactions were stirred at 200 rpm and 30 °C for 24 h, unless otherwise specified. To stop the reaction, 10% TFA (10 µL) was added to each well, and denatured protein removed by centrifugation (10 min, 12,000 rpm, 4 °C). The supernatant (100 µL) was diluted with a 50% solution of DMSO in water (900 µL), and analysed by analytical HPLC. The pH was varied by changing the pH of the KPi buffer (6.0 - 9.0). Reactions were also run at 50 °C to test the effect of temperature.

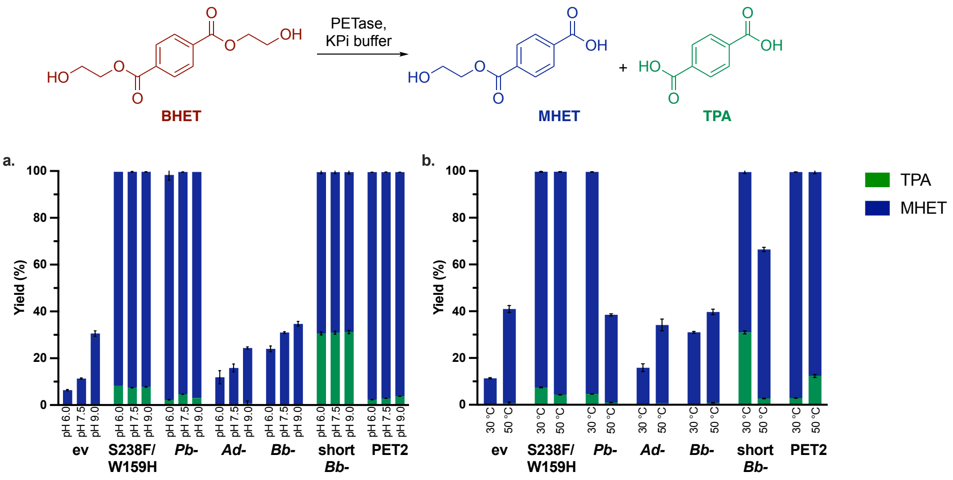

Figure S5. Effects of (**a.**) pH (6.0 - 9.0) and (**b.**) temperature (30-70 °C) on PETase (0.7-1.2 mg/mL) mediated hydrolysis of BHET (5 mM) to MHET and TPA in KPi buffer (50 mM) for 24 h with shaking (200 rpm). It should be noted here that MHET values for these two reactions are estimated from the quantity of remaining BHET starting material and of breakdown product TPA.

Scaled solution BHET powder degradation reaction. Reactions with BHET (200 mg, pre-crushed with pestle and mortar) were scaled up to 14 mL in KPi buffer (50 mM, pH 7.5). Whole cells (50 mg) were resuspended in KPi buffer (4 mL) prior to reaction. Reactions were left at 175 rpm for 8 h, at 30 °C. Reactions were stopped by freezing. Reactions were heated to 37 °C to defrost and sonicated for 2 x 5 min at 9 μ. Aliquots (500 μL) were diluted with H_2O (250 μL) and DMSO (750 μL). An aliquot was taken and diluted by a factor of 1000. Samples were then passed through a 0.45 μm centrifuge filter (13,200 rpm, 15 min, 18 °C). The supernatant was analysed by analytical HPLC.

Mechanoenzymatic BHET powder degradation reaction. BHET (200 mg, pre-crushed with pestle and mortar) was added to the jars, along with 2 x 5 mm stainless steel balls and KPi buffer (200 μL, 50 mM, pH 7.5). Whole cell enzyme (50 mg) was added and the reactions milled for 20 min per hour with 40 min aging, for 8 h total, at 30 Hz. Reaction jars were washed out with 2 x DMSO (3.5 mL) and H_2O (3.5 mL) alternately. Reactions were then frozen until analysis. Reactions were heated to 37 °C to defrost and sonicated for 2 x 5 min at 9 μ. Aliquots (500 μL) were diluted with H_2O (500 μL) and DMSO (500 μL). An aliquot was taken and diluted by a factor of 27. Samples were then passed through a 0.45 μm centrifuge filter (13,200 rpm, 15 min, 18 °C). The supernatant was analysed by analytical HPLC. Reactions were completed in duplicate.

PET powder degradation assay. Enzymatic reactions were carried out in 96-well plates containing clarified cell lysate (1.0 mg/mL), KPi buffer (50 mM, pH 7.5) and PET powder (4 mg/mL) in 200 μL total volume. Reactions were stirred at 700 rpm and 30 °C for 96 h. To stop the reaction, DMSO (200 μL) was added to each well and the reaction mixture (400 μL) was vortexed then filtered through a 0.45 μm centrifuge filter to remove undegraded PET substrate and protein (15 min, 13,200 rpm, 18 °C). The supernatant was analysed by analytical HPLC to determine the concentration of breakdown products (TPA, MHET and BHET). The pH was varied between 6.0 and 9.0. The temperature was varied between

30 °C and 50 °C. The concentration of PET in the reactions was varied between 1 mg/mL and 4 mg/mL. The total reaction time was varied between 24 h and 96 h. Reactions were completed in triplicate (Figure S6).

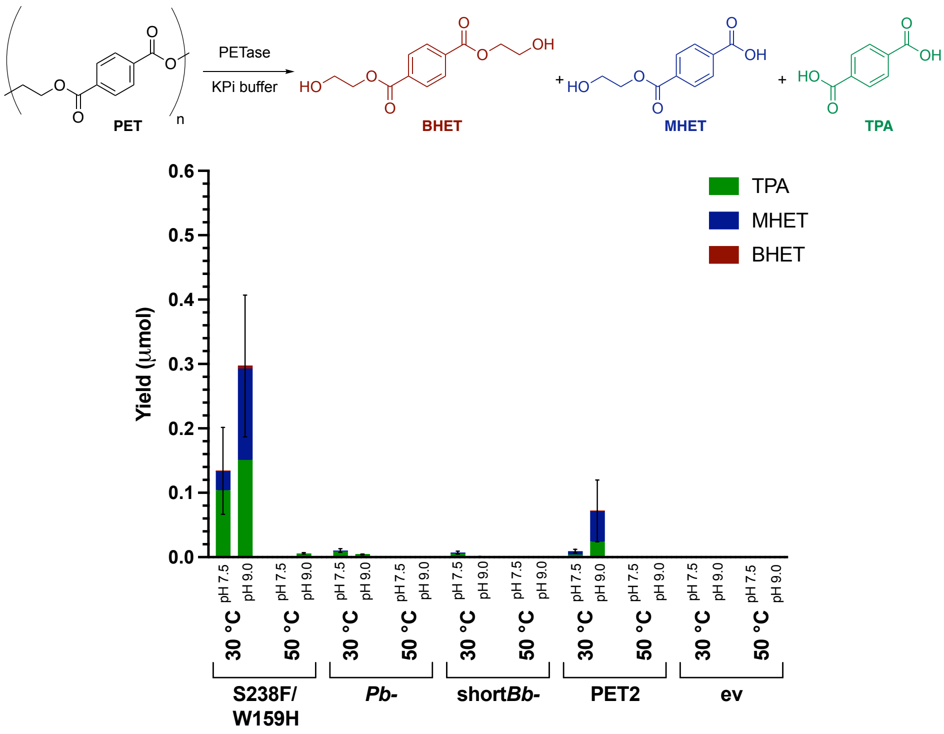

Figure S6. Small scale solution hydrolysis of PET powder. PET powder (4 mg/mL), clarified lysate (1.0 mg/mL) in KPi buffer (50 mM) for 96 h with shaking (700 rpm) testing the effect of temperature, by the four selected PETases.

Scaled solution PET powder degradation reaction (~4 mg/mL). Reactions with PET substrates (50 mg) were scaled up to 15 mL in KPi buffer (50 mM, pH 7.5). Clarified lysate was prepared fresh for use from whole cells (50 mg), as per enzyme preparation. Reactions were left at 160 rpm for 8 h, at 30 °C. Reactions were stopped by freezing. Reactions were heated to 37 °C and sonicated for 2 x 5 min at 9 µ. Aliquots (500 µL) were diluted with H_2O (250 µL) and DMSO (750 µL). Samples were then passed through a 0.45 µm centrifuge filter (13,200 rpm, 15 min, 18 °C). The supernatant was analysed by analytical HPLC. Reactions were completed in duplicate.

Scaled solution PET powder degradation reaction (~10 mg/mL). Reactions with PET substrates (50 mg) were scaled to 5 mL in KPi buffer (50 mM, pH 7.5). Clarified lysate was prepared fresh for use from whole cells (50 mg), as per enzyme preparation. Reactions were left at 160 rpm for 8 h, at 30 °C. Reactions were stopped by freezing. Reactions were heated to 37 °C and sonicated for 2 x 5 min at 9 µ. Aliquots (500 µL) were diluted with H_2O (250 µL) and DMSO (750 µL). Samples were then passed through a 0.45 µm centrifuge filter (13,200 rpm, 15 min, 18 °C). The supernatant was analysed by analytical HPLC. Reactions were completed in duplicate.

PET powder degradation with ball milling. PET powder was added to the jars, along with 2 x 5 mm stainless steel balls and KPi buffer (200 µL, 50 mM, pH 7.5). Whole cell enzyme (50 mg) was added and the reactions milled for 5-30 min per hour, for 8 h total, at 30 Hz. Reaction jars were washed out with 2 x DMSO (3.5 mL) and H_2O (3.5 mL) alternately. Reactions were then frozen until analysis. Reactions were heated to 37 °C to defrost and sonicated for 2 x 5 min at 9 µ. Aliquots (500 µL) were diluted with H_2O (500 µL) and DMSO (500 µL). Samples were then passed through a 0.45 µm centrifuge filter (13,200 rpm, 15 min, 18 °C). The supernatant was analysed by analytical HPLC. Reactions were completed in duplicate.

Other PET substrates degraded with ball milling. PET substrates (50 mg) were added to the jars, along with 2 x 5 mm stainless steel balls and KPi buffer (200 µL, 50 mM, pH 7.5). Whole cell enzyme (50 mg) was added and the reactions milled for 20 min per hour, for 8 h total, at 30 Hz. Reaction jars were washed out with 2 x DMSO (3.5 mL) and H_2O (3.5 mL) alternately. Reactions were then frozen until analysis. Reactions were heated to 37 °C to defrost and sonicated for 2 x 5 min at 9 µ. Aliquots (500 µL) were diluted with H_2O (500 µL) and DMSO (500 µL). Samples were then passed through a 0.45 µm centrifuge filter (13,200 rpm, 15 min, 18 °C). The supernatant was analysed by analytical HPLC. Reactions were completed in duplicate.

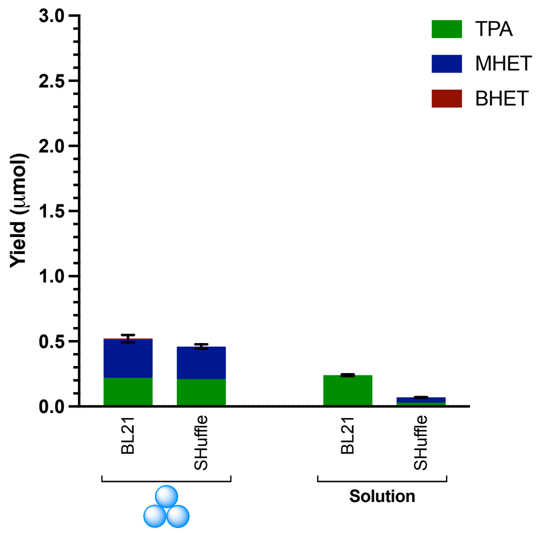

Figure S7. Mechanoenzymatic and solution degradation of PET powder (50 mg) using an empty vector of BL21 *E. coli* cells in comparison with an empty vector of *E. coli* SHuffle T7 cells. Optimised RAging cycles of T = 20 min milling and 40 min aging x 8 cycles, 30 Hz were used. Empty vector (ev) controls use predominantly BL21.

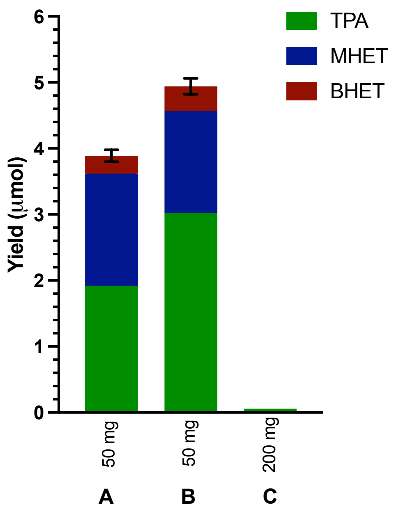

Figure S8. Mechanoenzymatic breakdown of PET powder, 30 Hz, 24 h total reaction time, using optimised RAging cycles of T = 20 min milling per hour and 40 min aging. Conditions are as follows: **A** 1 mg purified PET2, 50 mg PET powder; **B** 12.5 mg lysate PET2, 50 mg PET powder; **C** 50 mg lysate PET2, 200 mg PET powder.

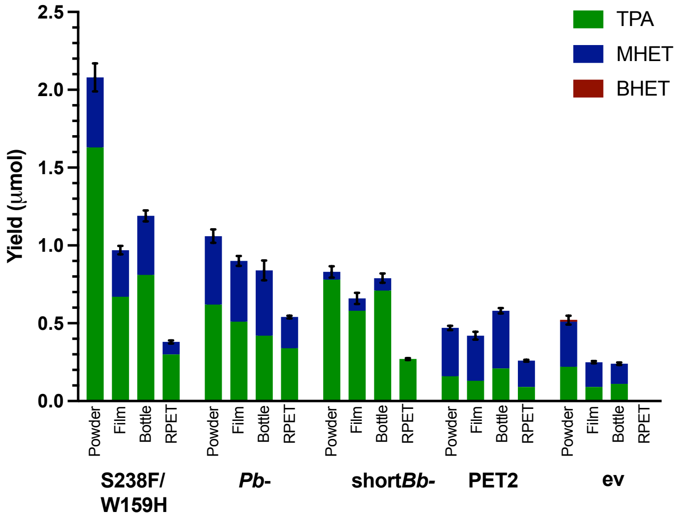

Figure S9. Mechanoenzymatic degradation of PET substrates. Displaying the same data as **Figure 4a.** with a comparison between substrates rather than PETases.

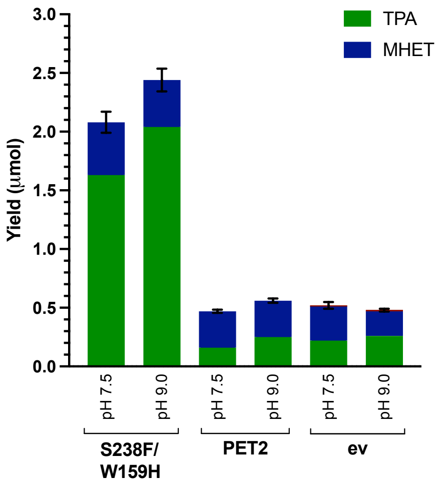

Figure S10. Mechanoenzymatic breakdown of PET powder using optimised RAging cycles of T = 20 min milling and 40 min aging x 8 cycles, 30 Hz, comparing the effect of pH change between pH 7.5 and 9.0 with *Is*-PETase S238F/W159H, PET2 and empty vector negative control.

2. Chemical synthesis

2.1 General Methods

^{1}H NMR and ^{13}C NMR spectra were recorded using a Bruker Avance Neo 700 MHz spectrometer at room temperature. The chemical shifts are recorded in parts per million (ppm), with the known residual solvent peaks corresponding to DMSO-d_6 (2.50 ppm). Coupling constants (J) are reported in Hz. The multiplicity of each signal is recorded as: s – singlet, t – triplet, dd – doublet of doublets, m – multiplet. NMR assignments use numbering independent from IUPAC. Low Resolution Mass Spectra was recorded on a Waters LCT Premier Q-TOF operating in negative ionisation mode.

2.2 Synthesis

Mono(2-hydroxyethyl)ester of terephthalic acid, MHET[4,5]

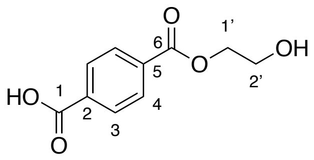

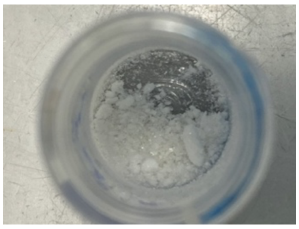

NaOH (0.16 g, 4.0 mmol) was added to a solution containing BHET (1.01 g, 4.00 mmol) dissolved in CH_3CN (25 mL) and H_2O (25 mL) and the reaction stirred for 16 h. The reaction was concentrated by rotary evaporation to ~10 mL, then HCl (2 M) was added until pH 2 - 3 was reached. H_2O (40 mL) was added and the resultant mixture centrifuged (4 °C, 5300 rpm, 20 min). The supernatant was evaporated to dryness. $CHCl_3$ (2 x 30 mL) and $NaHCO_3$ (30 mL), were added and then the layers separated. The pH of the aqueous layer was adjusted to ~pH 3 with HCl (6 M) and this was extracted with further $CHCl_3$ (3 x 30 mL). The aqueous layer was left for 9 days and white crystals formed which were analysed by ^{1}H NMR spectroscopy, confirming them to be **MHET** (46 mg, 5%). Crystals were lyophilised to remove traces of water. Analytical HPLC confirmed the purity of MHET in the sample as 97.04% (remaining 2.96% corresponded to TPA). Rf 0.26 (2:8, MeOH:CH_2Cl_2); m.p. 173-174 °C (H_2O) (lit. m.p. 178-188 °C);[4,5] ^{1}H NMR (700 MHz; DMSO-d_6) δ 13.34 (1H, br s , 1-OH), 8.10 – 8.04 (4H, m, 3-H_2, 4-H_2), 4.94 (1H, t, J = 5.7 Hz, 2’-OH), 4.30 (2H, t, J = 4.9 Hz, 1’-H), 3.71 (2H, dt, J = 5.7 and 4.9 Hz, 2’-H); ^{13}C NMR (176 MHz; DMSO-d_6) δ 166.6, 165.3, 134.9, 133.4, 129.6, 129.5, 67.0, 59.0; *m/z* [ESI-] 209 ($[M-H]^-$, 100%), 441 ($[2M-H+Na]^-$, 10%). All spectral data corresponds to those given in the literature.

2.3 NMR spectra

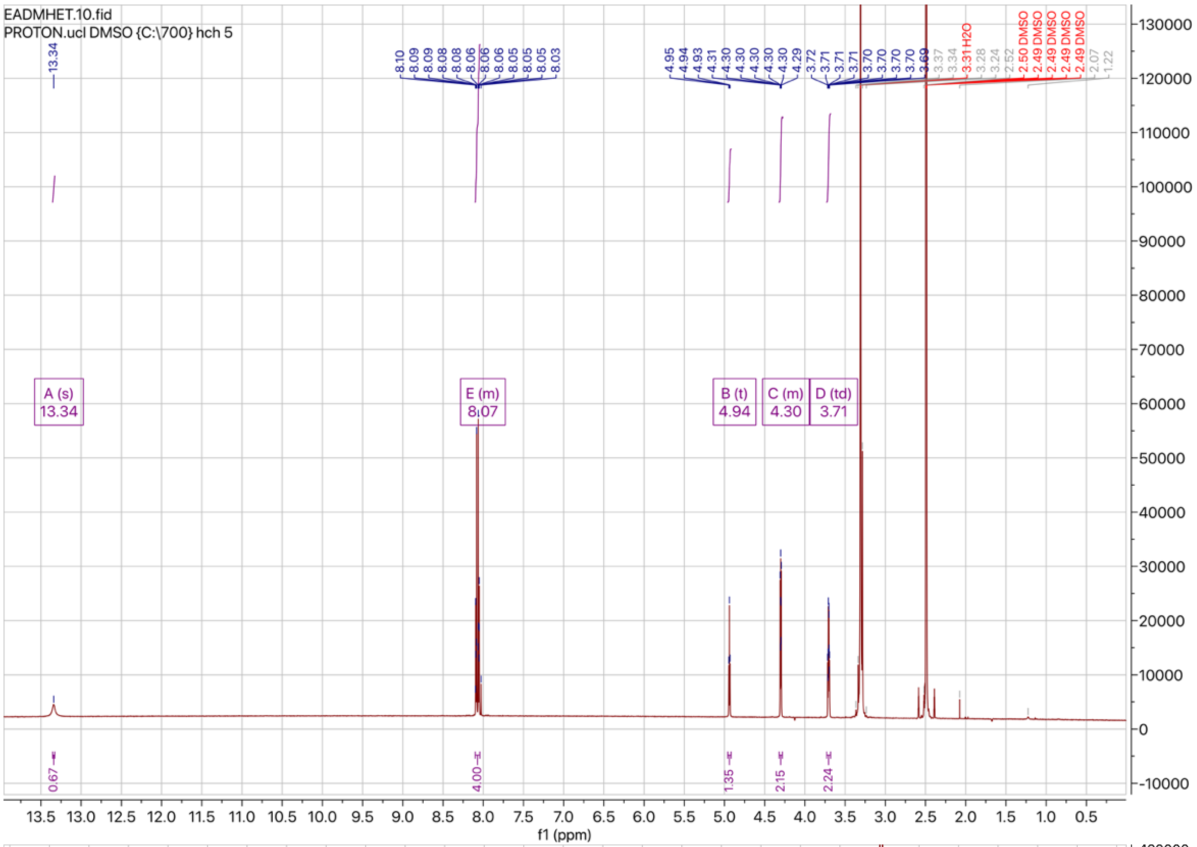

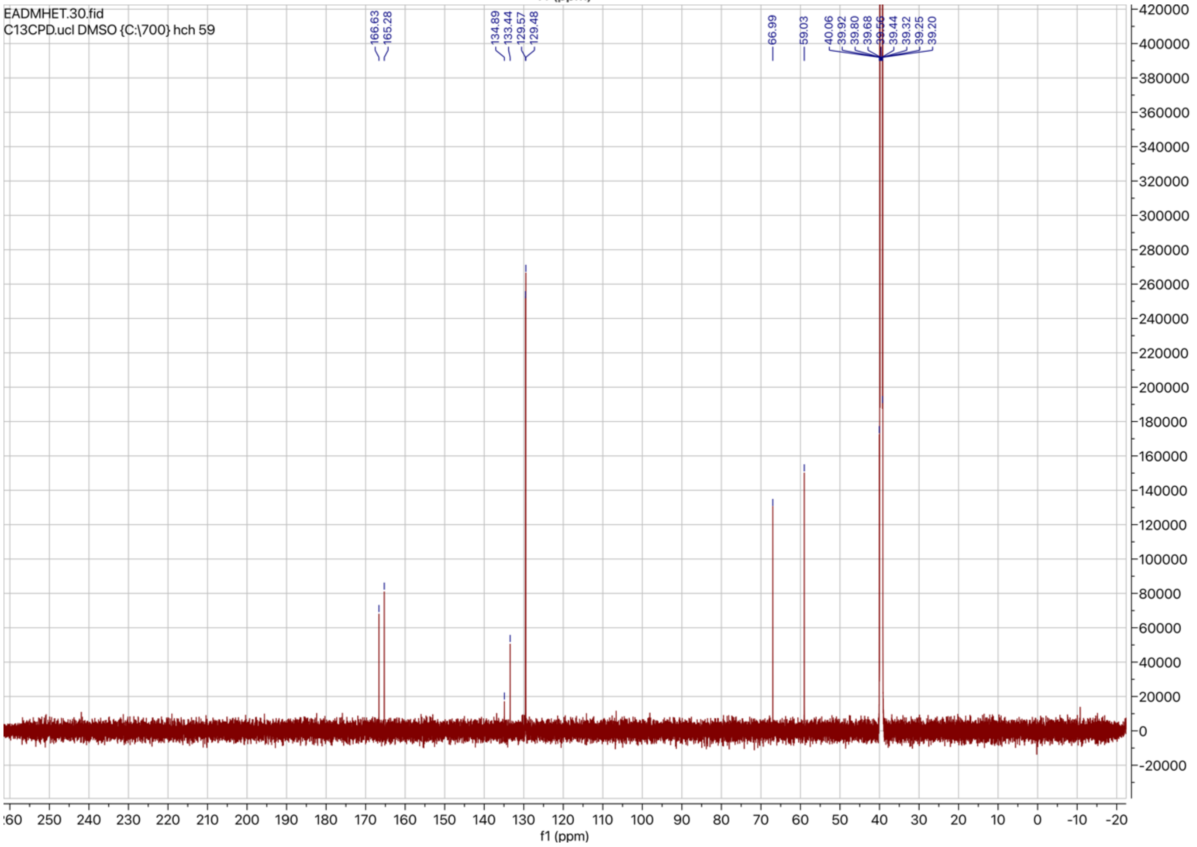

3. References

1 H. Y. Sagong, S. Kim, D. Lee, H. Hong, S. H. Lee, H. Seo and K. J. Kim, *J. Hazard. Mater.*, 2022, **429**, 128267.

2 C. C. Chen, X. Han, X. Li, P. Jiang, D. Niu, L. Ma, W. Liu, S. Li, Y. Qu, H. Hu, J. Min, Y. Yang, L. Zhang, W. Zeng, J. W. Huang, L. Dai and R. T. Guo, *Nat. Catal.*, 2021, **4**, 425–430.

3. E. L. Bell, R. Smithson, S. Kilbride, J. Foster, F. Hardy, S. Ramachandran, A. B. Tedstone, S. J. Haigh, A. B. Garforth, P. J. R Day J, C. Levy, M. P. Shaver and A. P. Green, *Nat. Catal.*, 2022, **5**, 673–681.

4. V. Tournier, C. M. Topham, A. Gilles, B. David, C. Folgoas, E. Kamionka, M. Desrousseaux, H. Texier, S. Gavalda, M. Cot, E. Guémard, M. Dalibey, J. Nomme, G. Cioci, S. Barbe, M. Chateau, I. André, S. Duquesne and A. Marty, *Nature*, 2020, **580**, 216–220.

5. G. J. Palm, L. Reisky, D. Böttcher, H. Müller, E. A. P. Michels, M. C. Walczak, L. Berndt, M. S. Weiss, U. T. Bornscheuer and G. Weber, *Nat. Commun.*, 2019, **10**, 1717.
